# Barriers, facilitators of sports participation and needs of South African Paralympians

**DOI:** 10.4102/ajod.v14i0.1532

**Published:** 2025-02-28

**Authors:** Siyabonga H. Kunene

**Affiliations:** 1 Department of Physiotherapy, Faculty of Health Sciences, University of the Witwatersrand, Johannesburg, South Africa; 2 Wits Sports and Health, Faculty of Health Sciences, University of the Witwatersrand, Johannesburg, South Africa

**Keywords:** barriers, facilitators, needs, athletes, sports, disability, inclusion, Paralympians

## Abstract

**Background:**

Despite the transformation initiatives, the inclusion of people with disabilities in sports remains a challenge. Athletes with disabilities (AWDs) in low- and medium-socioeconomic countries are still being left behind, including in South Africa. They are facing various challenges.

**Objectives:**

This study aimed to explore barriers and facilitators to sports participation and the needs of AWDs.

**Method:**

This was a qualitative study design based on semi-structured interviews. Interviews were conducted with South African Paralympians. Permission was obtained from a physical disability association. Ethical clearance was issued by the University of the Witwatersrand Human Research Ethics Committee. An interview schedule with predetermined questions was used to guide the interviews. Interviews were held face-to-face or online from 20 min to 30 min per interview. All participants gave consent. The data were transcribed verbatim and analysed in themes deductively.

**Results:**

A total of 23 athletes participated, 12 of which were females and 11 were males. Participants were mostly Africans (*n* = 23) with a mean age of 26 years. All had over 5 years of sporting experience. Barriers included: Social stigma, a lack of disability awareness, limited opportunities to participate in sports; limited access to resources and services. Facilitators included: health; belonging; fulfilment, winning, and support from loved ones.

**Conclusion:**

Results showed a need to scale up disability inclusion, especially regarding the rendering of healthcare services and making resources available.

**Contribution:**

This article provides knowledge that may be useful as a baseline for developing a suitable intervention for AWDs.

## Introduction

Sport is a form of physical activity with many health benefits including lowering risks of lifestyle diseases, for example, cardiovascular diseases, obesity, type 1 diabetes, and many other diseases (World Health Organization [WHO] 2024). People with disabilities (PWDs) benefit physically, mentally, and socially when participating in sports (United Nations 2011). Sports has positively changed the lives of several PWDs. It has improved the quality of life and it has the power to inspire greatness.

Unfortunately, PWDs usually spend most of their time indoors doing less active activities, for example, sleeping or watching television, etc. (Meyer & Mok 2019; Pagán-Rodríguez 2014). They are not involved in sports and cultural activities. As a result, they do not meet the minimum physical activity requirements of at least 150 min per week (WHO 2024). This is why their risks of lifestyle diseases will be high. Rehabilitation therapists have tried to incorporate sports as part of rehabilitation programmes for PWDs (Bragaru et al. 2011; Van Der Ploeg et al. 2007). Sports for PWDs started as a form of a rehabilitation programme and it has developed into what we know now as Paralympics. Sir Ludwig Guttman, a neurologist, was the most important figure in the development of sports for PWDs. He established the Stoke Mandeville Games, which evolved to the high level of sports participation that we see today (Ghosh & Bhowmick 2018).

People with disabilities face many barriers to sports participation including a lack of motivation, inaccessible facilities, a lack of time and opportunities, a lack of transportation, no access to information, and many more (Rimmer et al. 2004; Tenenbaum & Eklund 2007). Issues of inaccessible services, discrimination and stigma can also be barriers to sports participation among PWDs (Trani et al. 2020). These barriers can be personal, physical, environmental, social, economic, and political. These barriers have a way of compromising the quality of life among athletes with disabilities (AWDs) because they prevent them from getting involved in sports and cultural activities. There is a need to address barriers to sports participation for PWDs.

As much as it is important to understand barriers to sports participation, it is also important to understand facilitators. These are factors that encourage sports participation among PWDs. Studies have reported on various facilitators of sports participation for able-bodied people and PWDs. These facilitators are similar. They include benefits to health, passion, desire, fun, enjoyment, the opportunity to socialise, motivation, and many more (Craig et al. 2019; Jaarsma et al. 2013; Shihui et al. 2007; Tenenbaum & Eklund 2007; Wu & Williams 2001). These factors can also be personal, physical, environmental, social, economic, and political as seen with barriers.

Athletes with disabilities face various challenges and have many unmet needs. They are still left behind and not included as compared to able-bodied athletes. South African athletes and many other athletes in other low- to medium-income countries are still facing challenges and have unmet needs (Rademeyer 2017). Most of the problems they are still facing today were caused mostly by political issues in the past and present. The apartheid era negatively impacted the lives of PWDs in countries such as South Africa before 1994. People with disabilities, especially the black population, were discriminated against and not provided with needed services. Many of them were not allowed to participate in sports because of the colour of their skin and their disabilities. Post-apartheid, South Africa has seen a faster growth in sports participation and performance among PWDs, even faster growth than other sporting codes in able-bodied sports (Rademeyer 2017).

There is still more work to be carried out to address the challenges and needs of PWDs. There is a lack of research that reports on the challenges and needs of AWDs, especially in South Africa and other low- to medium-socioeconomic countries. The aim of this study was, therefore, to determine barriers and facilitators to sports participation and the needs of AWDs in South Africa.

## Research methods and design

### Study design

This was a qualitative study that used a semi-structured interview method to solicit responses from AWDs regarding barriers and facilitators to sports participation and needs.

### Participants

This study included Paralympians from various sporting codes including archery, athletics, swimming, and cycling. These were athletes who were affiliated with the South African Sports Association for the Physically Disabled (SASAPD). Paralympians were included in this study because of convenience (easy access to their database). These were athletes who represented South Africa in the Tokyo 2020 Paralympics.

### Recruitment

Before recruitment commenced, the author obtained ethical clearance from the University of the Witwatersrand Human Research Ethics Committee (No. M220120). Permission from SASAPD and various sporting codes was given in writing. The author recruited participants by first sending an email to SASAPD asking for the dissemination of the study information to their athletes who met the inclusion criteria. The author then scheduled interview sessions with those athletes who showed an interest in participating in the study. Participants consented in writing before the data collection process commenced.

### Data collection tool and procedure

An interview schedule was used to guide the data collection process. The author developed the interview schedule with questions relating to barriers and facilitators to sports participation and the needs of participants. The questions were informed by the international classification of functioning and health framework (Chan et al. 2009). The schedule was validated by five experts who were seasoned researchers in disability studies, especially qualitative studies. The schedule included the following structure: opening remarks and an introduction, outlining ground rules, questions, and a summary of the session. The interviews were conducted face-to-face or via the Zoom online platform. On the day of data collection, the author requested each participant to sign a consent form before the interview started. Participants were also asked to consent to the interview being recorded. Each interview session lasted for approximately 20–30 min. Data were collected to the point of data saturation. All recordings were kept in a password-protected hard drive.

### Data analysis

The recorded data were transcribed verbatim. The names of the participants were coded numerically in the transcripts. The data were analysed thematically, the process being inductive. The process of analysing data followed the following steps: (1) familiarisation of data by reading and re-reading the original transcript, while listening to the audio recording, (2) development of themes and subthemes from the concepts and categories from the data, and (3) defining and naming the themes and subthemes. The data analysis process involved a second reviewer. Several discussion rounds happened among reviewers, and they verified their findings with each other to test the credibility of the data search process.

### Ethical considerations

The study obtained ethical clearance from the University of the Witwatersrand Human Research Ethics Committee (No. M220120). Participants received a study information sheet and they consented in writing. They also consented to have the interviews voice recorded. No personal information (e.g. names, contact details, addresses, etc.) was collected from participants. Each participant was identified by a special code that was allocated to each participant. Voice recordings and transcription documents were stored in password-protected One Drive cloud space. Only the author and research assistant had access to the data.

## Results

### Demographics

A total of 23 athletes participated in the study (Table 1). Three athletes were from archery, 12 from athletics, five from swimming, and three from the cycling code. Most participants (*n* = 16) had 5–10 years of experience in sports. Types of impairments that participants had included were visual impairment, limb deficiency, paraplegia, quadriplegia and cerebral palsy.

**TABLE 1:** Demographic profile of participants (*N* = 23).

Characteristics	Categories	*n*	%
Gender	Male	11	48
	Female	12	52
Age (years)	15–17	2	9
	18–35	18	78
	36–45	3	13
Sporting codes	Archery	3	13
	Athletics	12	52
	Swimming	5	22
	Cycling	3	13
Years of sporting experience	5–10	16	70
	11–20	7	30
Type of impairments	Visual impairment	4	17
	Limb deficiency	7	30
	Paraplegia	5	22
	Quadriplegia	2	9
	Cerebral palsy	5	22

### Barriers

Table 2 shows four themes and 11 sub-themes that emerged from the collected data. The following paragraphs will describe these themes and sub-themes.

**TABLE 2:** Themes and sub-themes on sports participation barriers (*N* = 23).

Themes	Sub-themes
Social stigma	A lack of knowledge on how to include people with disabilities in sports
	Discrimination
	Negative attitudes
A lack of disability awareness	Misconceptions about disability among the general population and sports community
	A lack of knowledge on how to include people with disabilities in sports
Limited opportunities to participate in sports	Scarcity of platforms or programmes
	Lack of knowledge about available opportunities
Limited access to resources	Lack of funding (to afford travel, equipment and affiliations or registrations)
	Unavailability of community sporting facilities (especially in under-resourced communities)
	Available sporting facilities are not disability friendly
Limited access to services	Limited access to medical and rehabilitation services
	A lack of suitable coaching personnel
	A lack of transportation

#### Social stigma

Participants indicated that social stigma is one of their barriers to sports participation. They reported the experience of discrimination by members of their communities because of their disabilities. They reported that some people have negative attitudes towards them. One of the female participants said: ‘… we are humans also, being disabled does not mean we are less of human beings …’ (Participant 8, Female, 17 years old). Another participant said:

‘… we are not a disability, we [*are*] people who live with disabilities, I wish people could just understand that, including the other normal athletes. This is who we are, we are just having different abilities …’ (Participant 4, Male, 28 years old)

A male participant also mentioned the following, when asked about discrimination:

‘… yes definitely. During my sporting career, that is one of the things that are frustrating to see that even though the constitution makes mention that there must be no discrimination, but it is still happening …’ (Participant 18, Male, 33 years old)

#### A lack of disability awareness

The results also showed that there are still misconceptions about disability among the general population and also among the sports community. As a result, people lack knowledge on how to include PWDs in sports. One male participant said:

‘… people need to be educated about disability. I don’t think they know us. They think their own things about us …’ (Participant 20, Male, 35 years old)

#### Limited opportunities to participate in sports

Another barrier to sports participation is the limited opportunities for PWDs to participate in sports. Participants mentioned that there are fewer training and competition opportunities for PWDs in South Africa. One female said:

‘… some of my friends who have funding are the ones who get to go overseas to train and compete. Here in South Africa there are not many opportunities to compete …’ (Participant 6, Female, 28 years old)

This study also found that a lack of knowledge among athletes and coaches about available competition opportunities is what limits AWDs from participating. One participant said:

‘… I realised after watching the 2012 Paralympics that people with my disability can also play sports. After that year, I then started [*playing*] sports.’ (Participant 1, Male, 23 years old)

#### A lack of services and resources

A lack of needed services (e.g. healthcare services, lack of qualified coaches for specific disability sporting codes, and lack of transport) is another barrier to sports participation that was identified. A male participant said:

‘… someone like me with paralysis of legs, I need to get physio. Getting to the hospital is a challenge, someone must arrange transports for me to go there …’ (Participant 15, Male, 24 years old)

Another male archery athlete indicated that he cannot afford rehabilitation services when he happens to sustain an injury: ‘… getting rehab is not easy for us. You physios are expensive …’ (Participant 9, Male, 43 years old). Most of the participants indicated that they do not have medical aids or personal finances to afford the required services. The little money they get from federations or national sports department seems not to be enough.

A lack of funding to buy equipment, travel, and access services seemed to be a major barrier to sports participation among AWDs. Most elite AWDs do not have sponsors and medical insurance. One lady participant said: ‘… sponsors don’t want to identify with us, maybe that is why we don’t get funding …’ (Participant 5, Female, 17 years old). Another female also said:

‘… what I can say is that there isn’t any funding. I’m very careful when I say that. There is funding, but the funding is not accessible to everybody. For instance, the funding I received, I received because I was in the top five in the World, so that was my privilege that I had …’ (Participant 12, Female, 31 years old)

Another one said: ‘… money is everything I need, everything needs money. I missed many opportunities because of lacking funding …’ (Participant 11, Female, 27 years old). One of the participants said: ‘… [*i*]t is difficult to see a physio because of a lack of funds. I would see a physio properly when we travel for big games.’ (Participant 3, Male, 34 years old).

Participants reported that a lack of sponsorships is one of the major barriers. Most participants indicated that even the workforce is not inclusive of PWDs. One runner mentioned: ‘… [*i*]t would be better if I was working because I can pay for my travelling and servicing of my artificial legs …’ (Participant 1, Male, 23 years old).

The unavailability and inaccessibility of sporting facilities in communities, especially low socioeconomic communities is a notable sports participation barrier for AWDs. Most available facilities are not disability friendly. A male swimmer said:

‘… swimming pools for training and competition are very scarce in my areas and the whole of South Africa. How are we then expected to compete internationally …?’ (Participant 4, Male, 28 years old)

Male archery also said: ‘… [*i*]’m lucky because I do training here on my farm. Archery training ranges are very few, some guys come to my farm for training …’ (Participant 7, Male, 45 years old).

#### Healthcare services

It was evident that participants had problems in accessing and affording healthcare services. The biggest issue was funding. Participants strongly expressed their need for medical and rehabilitation services to deal with their existing conditions and future injuries and illnesses. One female mentioned the following when asked about her needs:

‘… my disability requires me to have medical treatment now and then. My financial situation sometimes prevents me from accessing medical services …’ (Participant 2, Female, 37 years old)

Another participant commented about a need for physiotherapy. He said:

‘… [*at*] least one physio session will help with my issues of stiffness and for general body maintenance …’ (Participant 13, Male, 30 years old)

#### Coaching

One swimmer said: ‘… coaches are available but they just need the right skills to deal with disabled athletes …’ (Participant 10, Female, 24 years old). There were many similar comments from participants that showed a need for specialised coaching. Another participant said:

‘… yes we do have coaching, but obviously it takes a little bit of reading up about disabilities and how to do adaptive training and so forth, but I think coaching a disabled athlete is a skill that a coach needs to learn, from the get-go …’ (Participant 13, Male, 44 years old)

#### Awareness

Another need that was identified was a need for community awareness about sports and disability. Participants felt that the public community still lacks knowledge about what disability is all about and how to include PWDs in sports. A participant from athletics said:

‘… some people assume things about us. I just feel people lack knowledge about disability. Maybe we should do something to educate them about who we are and what we need.’ (Participant 23, Male, 30 years old)

Access to information by AWDs was also identified as a need. Most participants indicated that they need to know where to access information about disability sports information. One cyclist mentioned the following: ‘… I honestly didn’t know anything about cycling sport for people with disabilities. I found out about it by chance …’ (Participant 21, Male, 36 years old).

### Facilitators

Participants were also asked about facilitators of sports participation. Various themes and sub-themes emerged from the collected data (Table 3).

**TABLE 3:** Themes and sub-themes on sports participation facilitators (*N* = 23).

Themes	Sub-themes
Good health	Physical health (to manage the existing condition and reduce risks of further disability)
	Non-physical health (managing mental health issues and emotional challenges)
Sense of belonging	Friendships that are built
	Having a special community
Fulfilment	The fun experiences
	Finding a purpose in life
Winning	Personal achievement
	Receiving performance prizes
	Personal drive
Support from friends, partners and family	The encouragement that is received
	Assistance provided

#### Good health

The benefit of good health is what facilitated sports participation in most participants. Many mentioned that sports as a form of exercise benefited their physical and non-physical health. Someone said:

‘… my involvement in sports started as part of rehabilitation for my condition. Running helps to improve my quality of health. That is why I do what I do …’ (Participant 19, Male, 38 years old)

Another participant from athletics mentioned the role of sports in dealing with mental health and emotional issues. She said:

‘… [*w*]hen I could not move my legs after surgery, I didn’t have a purpose for living, but getting involved in swimming helped me a lot with stress and it made me happy again …’ (Participant 17, Female, 31 years old)

#### Sense of belonging

The results showed that participants felt a sense of belonging when they participated in sports. They enjoyed making friends and belonging to a community. This facilitated sports participation. An archery athlete said: ‘… the friends I found in archery motivate me to do my sport. Without my friend Shaun, I don’t know where I would be …’ (Participant 16, Male, 43 years old). Another participant from athletics said: ‘… sports is about friendship. It creates a healthy social environment. It makes me feel normal again …’ (Participant 23, Male, 30 years old).

#### Fulfilment

The fun and a sense of purpose were reported as facilitating sports participation among participants. It gave participants fulfilment. One long jumper said:

‘… what encourages me to do sport is the fun and happiness that I get when I’m with my friends who care for me and my wellbeing. I just love it, mostly for that reason …’ (Participant 8, Female, 17 years old)

Another participant said sports gave him a reason to exist. He said: ‘… doing sport is one thing that gives me purpose in life. Yes, I do work, but sports is what I enjoy more …’ (Participant 3, Male, 34 years old).

#### Winning

Personal achievement and winning prizes were also reported to be motivating athletes to participate in their sports. A shot-put athlete said: ‘… that feeling you get when you win boosts you big time bro, you don’t understand …’ (Participant 9, Male, 43 years old). Another participant also added: ‘… seeing yourself making progress, getting your PB (personal best) motivates me a lot …’ (Participant 1, Male, 23 years old). Personal drive is another facilitator that came up. One participant said:

‘… I had always participated in sports and I’ve always loved sports and I’ve done athletics and rugby and I’ve participated with able-bodied athletes and my disability (below elbow amputee) made it possible because the severity is not that big, I can still participate against able-bodies athletes. But maybe what made it easy for me was my drive.’ (Participant 11, Female, 27 years old)

Another one said: ‘… the first thing is that will inside of you.’ (Participant 12, Female, 31 years old).

#### Support from friends, partners, and family

Having supportive friends, partners, and family was also reported as a big facilitator of sports participation. It was reported that support from friends, partners, and family played a big role to encourage and provide personal assistance. One participant said:

‘… its people around me, my family, friends, and my boyfriend that motivate me and give me support … They encourage me and help me with everything I need.’ (Participant 10, Female, 24 years old)

One runner also said:

‘… my parents who adopted me played a big role for me to be here. They helped me get new legs (artificial legs) and paid people to teach me how to run …’ (Participant 4, Male, 28 years old).

## Discussion

Sports play a positive role in the lives of PDs. It improves health, well-being, resilience, and social support for them (Mira et al. 2023). Sports seem to provide a good setting where PWDs can reproduce unhelpful disablist discourses (Swartz et al. 2018). The results of this study reported various sports participation facilitators. These are factors that encouraged or motivated AWDs to participate in sports. These factors included health benefits, a sense of belonging, fulfilment, winning, and support from loved ones. Various studies reported similar facilitators, which included health benefits, passion, desire, fun, enjoyment, the opportunity to socialise, motivation, support from friends and family, achievement of goals, empowerment and advocacy, college scholarships, and more (Craig et al. 2019; Jaarsma et al. 2014; McLoughlin et al. 2017; Shihui et al. 2007; Tenenbaum & Eklund 2007; Wu & Williams 2001). All these facilitators encourage or motivate PWDs to get involved in sports and stay in sports to enjoy the benefits that come with participation.

The results also showed numerous sports participation barriers. These barriers included issues of discrimination, stigma, a lack of opportunities, poor access to key services, limited access to resources, a lack of access to facilities, and a lack of knowledge. The challenges reported in this study were personal, physical, environmental, social, economic, and political. These are similar challenges identified among AWDs in the United Kingdom (UK) (Ives et al. 2021). In their semi-structured interview study, Aves et al. reported that participation was hampered by many extrinsic and intrinsic barriers. The extrinsic barriers included material issues. These issues included poor access to facilities, activities, and services (physical barriers); issues of cost and affordability (economic barriers), and issues of funding and unavailability of services (political barriers). Internal barriers included fear of the unknown, lack of self-esteem, feelings of being judged, and many other personal issues (e.g. perceived lack of enjoyment). In another focus group study, Rimmer et al. (2004) reported a multifactorial set of similar barriers to sports participation. These barriers ranged from environmental, economic emotional, and psychological, equipment-related, personal, and political issues as well.

Another South African study described how AWDs shared their experiences of involvement in competitive sports for PWDs (Swartz et al. 2018). It was an in-depth semi-structured study conducted among 20 athletes. Their participants described complex and contradicting experiences that included some positive and negative experiences. These contradictions highlighted political and ideological tensions relating to the inclusion and representation of PWDs. All these challenges show how serious the issues of having a disability are. They show how challenging it is for PWDs to enjoy sports as compared to able-bodied athletes.

The results of this study also reported the needs of AWDs, which included personal funding, accessible and affordable healthcare services, the need for suitable coaching personnel, and awareness.

Despite the transformation agenda to ‘Leave No One Behind’ by the United Nations, PWDs are still experiencing many challenges and have many unmet needs (United Nations 2011). People with disabilities are still left behind because of the above-mentioned barriers and unmet needs. According to Trani et al. (2020), PWDs are still facing issues of stigma, discrimination, poverty, unemployment, and poor delivery of needed services (e.g. health and education services). There is a need to address social stigma, discrimination, and all the identified barriers to sports participation including issues of access and affordability of services, especially in countries such as South Africa. A robust improvement in the way healthcare services are delivered is critical. A change is critical to ensure that PWDs are not left behind. It is necessary to ensure well-being, reduce inequality in all its dimensions, promote inclusion, and ensure availability and affordability of services.

There is a need for an intervention that will challenge barriers and cater to the needs of AWDs, especially in countries such as South Africa (Kunene 2023). There is a need to think beyond disability and focus on abilities, thus eliminating stigma and discrimination. People with disabilities must be fully included in communities and not feel discriminated and ostracised. Everyone has a role to play, including those in positions of authority, those in the healthcare sector, those in the sports sector, those in business, communities, families, and friends. Funding is needed, healthcare services are needed, suitable coaching personnel are needed, and awareness must be improved. Working together is what will make a difference. There is a need for a robust discussion among relevant stakeholders regarding suitable intervention strategies. A model of care is needed to improve the way healthcare and other services are delivered.

## Conclusion

This study aimed to explore barriers and facilitators to sports participation and the needs of AWDs in South Africa. Numerous barriers to sports participation were explored. These included discrimination, stigma, a lack of opportunities, poor access to key services, limited access to resources, a lack of access to facilities, and a lack of knowledge. Facilitators included health, belonging, fulfilment, winning, and support from loved ones. Needs included funding, healthcare services, coaching, and awareness. There is a need to scale up disability inclusion, especially regarding the rendering of healthcare services and making resources available. There is also a need to think beyond disability, have a robust discussion, challenge barriers and social attitudes, and develop and implement a suitable model of care. Everyone has a role to play in changing the status quo.
